# Convex Optimization via Symmetrical Hölder Divergence for a WLAN Indoor Positioning System

**DOI:** 10.3390/e20090639

**Published:** 2018-08-25

**Authors:** Osamah Abdullah

**Affiliations:** Department of Electrical Power Engineering Techniques, Al-Ma’moun University College, Baghdad 00964, Iraq; osamah.abdullah@wmich.edu

**Keywords:** information geometry, centroid, Bregman information, Hölder divergence, indoor localization

## Abstract

Modern indoor positioning system services are important technologies that play vital roles in modern life, providing many services such as recruiting emergency healthcare providers and for security purposes. Several large companies, such as Microsoft, Apple, Nokia, and Google, have researched location-based services. Wireless indoor localization is key for pervasive computing applications and network optimization. Different approaches have been developed for this technique using WiFi signals. WiFi fingerprinting-based indoor localization has been widely used due to its simplicity, and algorithms that fingerprint WiFi signals at separate locations can achieve accuracy within a few meters. However, a major drawback of WiFi fingerprinting is the variance in received signal strength (RSS), as it fluctuates with time and changing environment. As the signal changes, so does the fingerprint database, which can change the distribution of the RSS (multimodal distribution). Thus, in this paper, we propose that symmetrical Hölder divergence, which is a statistical model of entropy that encapsulates both the skew Bhattacharyya divergence and Cauchy–Schwarz divergence that are closed-form formulas that can be used to measure the statistical dissimilarities between the same exponential family for the signals that have multivariate distributions. The Hölder divergence is asymmetric, so we used both left-sided and right-sided data so the centroid can be symmetrized to obtain the minimizer of the proposed algorithm. The experimental results showed that the symmetrized Hölder divergence consistently outperformed the traditional k nearest neighbor and probability neural network. In addition, with the proposed algorithm, the position error accuracy was about 1 m in buildings.

## 1. Introduction

The global positioning system (GPS) is the world’s most utilized location system, but it cannot be used to accurately identify indoor locations due to the lack of line-of-sight between GPS receivers and satellites. Smartphones can provide location-based services in pervasive computing; they bring the power of GPS inside buildings. A previous study [1] showed that the global indoor positioning market is expected to grow from $935.05 million in 2014 to approximately $4.42 billion in 2019, corresponding to compound annual growth rate of 36.5%. Many technologies have been used instead of GPS, such as radiofrequency identification, Bluetooth, magnetic field variations, ultrasound, light-emitting diode light bulbs, ZigBee, and WiFi signals, to create high-accuracy indoor localization-based systems. These technologies are considered from a cost perspective.

With the widespread use of smart phones in the past decade, there has been an increasing demand to use indoor positioning systems (IPSs) to determine the position of objects and people inside buildings. In general, there are trade-offs between cost and an IPS technology. For example, ultrasonic technology has high accuracy but is also costly due to the large installation required. Since deployment of the WiFi infrastructure, it has been widely used to estimate the position of an object. The received signal strength (RSS) is a metric value that can be obtained from existing WiFi access points (APs) by any device equipped with a WiFi network adapter. The WiFi infrastructure does not require installation costs or specific hardware [2,3]. Nevertheless, IPSs face many challenges in indoor environments due to the unique properties and transient phenomena such as multipath propagation and signal attenuation. Signal attenuation is caused by people, furniture, and walls, which can limit the ability to design an accurate positioning system [4,5].

IPSs can be classified into two main categories: fingerprint-based techniques and log-distance propagation model algorithms, the latter of can be divided into angulation and lateration methods. Lateration methods calculate the absolute or relative position of an object by measuring distances from multiple reference points using geometry information such as angle of arrival, time of arrival, and time difference of arrival from the signals of APs. However, lateration-based techniques suffer from inaccurate location estimation; for example, it was reported in Reference [6] that the average localization distance error is 24.73 ft with a width of 80 ft and a length of 200 ft in a typical office scenario. Such inaccurate estimations occur for two reasons: non-line-of-sight propagation and inaccurate calculation of one or more of the APs’ axes. Thus, fingerprinting-based localization has become the more dominant technique in IPSs and has two major phases. First, the offline phase, in which the RSS value is recorded with their coordinates at predetermined reference points (RPs) to generate a radio map database [7,8,9].

The k nearest neighbor (kNN) is one simple way to estimate the location of an object by using the Euclidean distance to estimate the dissimilarity between the offline and online phases. The kNN algorithm has low accuracy and is easy to implement compared to other algorithms, such as Bayesian modeling and statistical learning, which have been used to estimate the location of an object. The localization distance error is one of the most fundamental metrics that determine the accuracy and reliability of the system. Variation in WiFi signals is an important issue [10,11]. There are several factors that affect WiFi signal propagation such as human bodies, radiofrequency (RF) equipment, and physical obstructions. These factors cause multiple issues, such as multipath wave propagation and signal attenuation, which can decrease the accuracy of the localization system [12].

The values stored in data maps represent the mean value of the RSS. Some approaches presume that the RSS distribution is Gaussian [13], whereas others presume non-Gaussian distributions [14]. Nevertheless, WiFi-based indoor localization systems have many advantages such as low cost and availability. Different hardware can significantly affect the accuracy of IPSs; for instance, it was reported in Reference [12] that RSS values collected using different smartphones at the same time and same location had different values. Furthermore, the orientation of the body can also contribute to the variance of the RSS signal; thus, the human body can be a significant signal attenuator.

In this paper, we use the Hölder divergence, which generalizes the idea of divergence in information geometry by smooth the non-metric of statistical distances in a way that are not required to follow the law of indiscernibles. The inequality of log-ratio gap pseudo-divergence is built to measure the statistical distance of two classes based on Hölder’s ordinary divergence. By experiment, the WiFi signal suffers from multimodal distribution; nevertheless, the Hölder divergence is considered the proper divergence to measure the dissimilarities between probability densities since the Hölder divergence is a projective divergence that does not need the distribution be normalized and allows the closed form expressions when the expansion family is an affine natural space like multinomial distributions.

Hölder divergences encompass both the skew Bhattacharyya divergences and Cauchy–Schwarz divergence and can be symmetrized, and the symmetrized Hölder divergence outperformed the symmetrized Cauchy–Schwarz divergence over the dataset of Gaussians. Both Cauchy–Schwarz divergences are part of a projective divergence distance family with a closed-form expression that does not need to be normalized when considering closed-form expressions with an affine and conic parameter space, such as multivariate or multinomial distributions.

The fingerprinting-based localization has two phases, the off-line phase and the on-line phase. In the off-line phase, we propose a procedure with a high characterization distribution. The RSS values were taken from four different orientations (45°, 135°, 225°, and 315°) to prevent body-blocking effects, with a scan performed for 100 s in each direction to reduce the effects of signal variation.

The fingerprinting radio-maps were decomposed into many clsuters using k-means-Bregman. The symmetrized k-means-Bregman showed unique results; the left-side centroid is the same Jensen–Shannon information radius as the right-side centroid that generalized the mean value of the cluster. Nevertheless, the right-side centroid was independent and always coincided with the center of the mass of the cluster point set. The symmetrized k-means-Bregman can be geometrically interpreted as a unique intersection of the linking between the two-sided centroid and the mixed-type bisector, and that generalized the two-sided centroid for a symmetrized k-means-Bregman.

## 2. Related Work

Most research on WiFi fingerprinting localization algorithms has focused on improvements in collecting fingerprinting data, which can decrease localization distance error and improve accuracy. Different algorithms have been proposed, some of which use the propagation properties of the signal, others that use ray tracing [15], and still others that use crowdsourcing-based inertial sensor data and indoor WiFi signal propagation models. Fingerprint-based location methods suffer from time variation between the offline and online phases. kNN is considered a pioneer algorithm that is used in localization-based algorithms. It uses the Euclidean distance to measure the similarity and dissimilarity between runtime and training data, after which the distance is sorted in increasing order. Some researchers use clustering techniques to reduce the impact of time variation by clustering the fingerprinting radio map into multi-partitions, after that the cluster that has lowest RSS-based distance will be chosen [15].

The cluster filtered kNN method was proposed in Reference [16] to partition the fingerprint radio map using hierarchical clustering; the proposed algorithm showed some improvement in the results. To improve the accuracy of the positioning system, Altintas and Serif [17] replaced the k-means algorithm with hierarchical clustering, which led to some improvement in the localization distance error. Likewise, it was proposed to incorporate kNN information into the fuzzy c-means clustering algorithm, so that a cluster could be chosen that matches an object’s location to estimate its location; the proposed algorithm resulted in little improvement in localization distance error within 2 m [18]. In Reference [19], affinity propagation was proposed with the coarse positioning algorithm to cluster the off-line of the database; the coarse algorithm works within one or more clusters to estimate the location of the object.

A new idea was proposed in Reference [20] by using a probabilistic distribution measurement, using a Bayesian network as a probabilistic framework to estimate the object’s location. The authors in Reference [21] proposed a modified probability neural network to estimate the location of the object, and this method outperformed the lateration technique. The authors in Reference [22] used a histogram of the RSS as a kernel method to estimate the object’s location. In Reference [23], the Kullback–Leibler divergence (KLD) algorithm was proposed to estimate the probability density function (PDF) as a composite hypothesis test between the test point and fingerprinting radio map, whereas in Reference [24], to estimate the location of the object, the authors assumed that the RSS had a multivariate Gaussian and used the KLD algorithm to estimate the PDF impact of the test point on the fingerprinting radio map. In Reference [25], a low energy RSS-based Bluetooth technique was proposed to create a radio map for fingerprinting, after which probabilistic kernel regression based on the KLD was used to estimate the location of the object. The localization distance error was approximately 1 m in an office environment.

## 3. Overall Structure of the IPS

**Figure 1:**
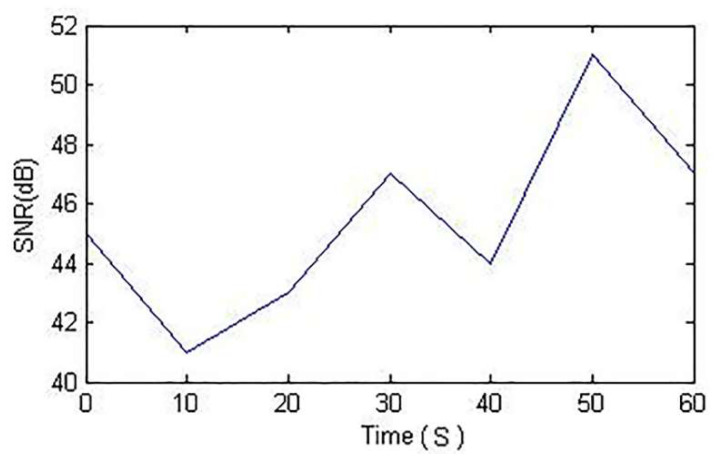
Signal-to-noise ratio of received strength signal indicator variations over time.

A typical WiFi fingerprint-based localization scenario was performed, in which a person held a smartphone device that had WiFi access, which was used to collect RSS measurements from different APs at various locations within the College of Engineering and Applied Sciences (CEAS) at Western Michigan University (WMU). As mentioned in Reference [26], an RSS distribution from multiple APs as a multimodal distribution commonly occurs. In our study, the signal-to-noise ratio was recorded for 35 min in a long corridor for a single AP. The mobile robot would stop every five minutes at each location and move 4 m further, and these steps were repeated for seven locations. We noticed values that differed by as much as 10 dBm, as shown in Figure 1.

There are many parameters that can affect the distribution of a signal such as diffraction, reflection, and pedestrian traffic [27]. We looked for a scenario that would lead to a better distribution of the AP signals. During the offline phase, a realistic scenario was performed that took signal variation into account. Because the human body can be an obstacle for signals, including the person holding the phone and the pedestrian in traffic, the fingerprint radio map was recorded from four different directions (45°, 135°, 225°, and 315°). At each RP, the RSS data were collected within the time sample, which was denoted as $\left\{q_{i,j}^{\left({}^{\circ}\right)}\left(\tau \right),\;\tau =1,\cdots ,t,\;t=100\right\}$, where (°) is the orientation direction and *t* represents the number of time samples. The covariance matrix and average of the RSS were calculated from four different directions, and 10 scans were used to create the radio map of the fingerprinting database, as represented by $Q^{\left({}^{\circ}\right)}$ [28]:

(1) $$Q^{\left({}^{\circ}\right)}=\left(\begin{array}{cccc} q_{1,1}^{\left({}^{\circ}\right)} & q_{1,2}^{\left({}^{\circ}\right)} & \cdots & q_{1,N}^{\left({}^{\circ}\right)}\\ q_{2,1}^{\left({}^{\circ}\right)} & q_{2,2}^{\left({}^{\circ}\right)} & \cdots & q_{2,N}^{\left({}^{\circ}\right)}\\ \vdots & \vdots & \ddots & \vdots \\ q_{L,1}^{\left({}^{\circ}\right)} & q_{L,2}^{\left({}^{\circ}\right)} & \cdots & q_{L,N}^{\left({}^{\circ}\right)} \end{array}\right)$$

where $q_{i,j}^{\left({}^{\circ}\right)}=\frac{1}{q}\overset{t}{\underset{t=1}{\sum }}q_{i,j}^{\left({}^{\circ}\right)}\left(\tau \right)$ and *t* = 10, which were arbitrarily chosen from 100 time samples. This can help us calculate the average value of RSS data over time for different APs, i=1,2,⋯,L, j=1,2,⋯,N, where *L* is the number of APs and *N* represents the number of RPs. The variance vector of each RP can be defined as:

(2) $$\Delta_{j}^{\left({}^{\circ}\right)}=⎣\Delta_{1,j}^{\left({}^{\circ}\right)},\Delta_{2,j}^{\left({}^{\circ}\right)},\Delta_{3,j}^{\left({}^{\circ}\right)},\ldots .\Delta_{L,j}^{\left({}^{\circ}\right)}⎦$$

where

(3) $$\Delta_{i,j}^{\left({}^{\circ}\right)}=\frac{1}{t-1}\sum_{\tau =1}^{t}{\left(q_{i,j}^{\left({}^{\circ}\right)}\left(\tau \right)-q_{i,j}^{\left({}^{\circ}\right)}\right)}^{2}$$

where $\Delta_{i,j}^{\left({}^{\circ}\right)}$ is the variance for AP *i* at RP *j* with orientation (°); thus, the database table of the radio map is $\left(x_{j},y_{j,}q_{j}^{\left({}^{\circ}\right)},\Delta_{j}^{\left({}^{\circ}\right)}\right)$ with $q_{j}^{\left({}^{\circ}\right)}$ defined as:

(4) $$q_{j}^{\left({}^{\circ}\right)}=⎣q_{1,j}^{\left({}^{\circ}\right)},q_{2,j}^{\left({}^{\circ}\right)},q_{3,j}^{\left({}^{\circ}\right)},\ldots .q_{L,j}^{\left({}^{\circ}\right)}⎦$$

During the online phase, the RSS measurement is denoted as:

(5) $$p_{r}=⎣p_{1,r},p_{2,r},p_{3,r,\ldots \ldots .}p_{L,r}⎦$$

## 4. Bregman Divergence Algorithm Formulation

The heterogeneity of RSS data makes it difficult to design IPSs with high accuracy that are dependent on fingerprinting-based locations. Indeed, the L_p_-norm and usual Euclidean distance do not always lead to IPSs with the highest accuracy, especially for systems with various histograms and other geometric features. It has been shown that using the information-theoretic relative entropy, known as the KLD, can lead to better results [29]. Bregman divergence has become a more attractive method for measuring similarity/dissimilarity between classes because it encapsulates the geometric Euclidean distance and information-theoretic relative entropy. The Bregman divergence $D_{F}$ between two sets of data, *p* = (*p*_1_, …, *p*_d_) and *q* = (*q*_1_, …, *q*_d_), and that associated with F (defined as a strictly convex function) can be defined as:

(6) $$D_{F}\left(p,q\;\right)=F\left(p\right)-F\left(q\right)-\langle \nabla F\left(p\right),p-q\rangle$$

where 〈..,..〉 denotes the dot product:

(7) $$\langle p,q\rangle =\sum_{i=1}^{d}p^{\left(i\right)}q^{\left(i\right)}=p^{T}q$$

and ∇F(p) denotes the gradient decent operator:

(8) $$\nabla F\left(p\right)={\left[\frac{\partial F}{\partial p_{1}}\ldots .\frac{\partial F}{\partial p_{d}}\right]}^{T}$$

**Figure 2:**
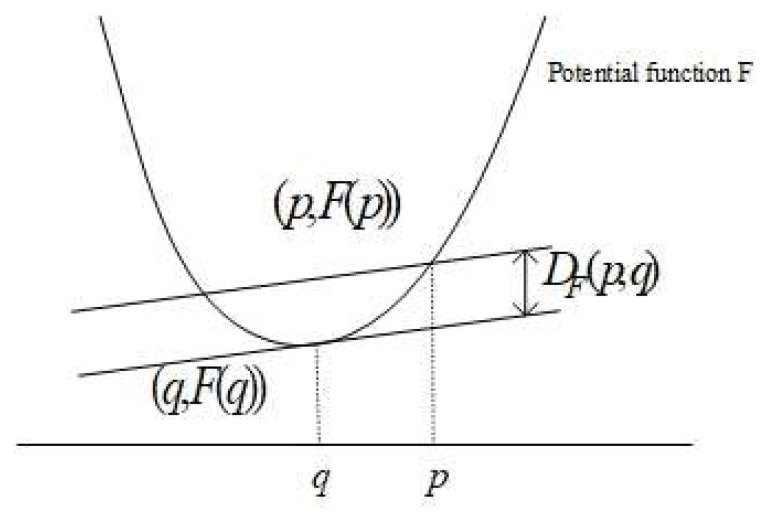
The Bregman divergence represents the vertical distance between the potential function and hyperplane at *q*.

The Bregman distance unifies the KLD with the Euclidean distance by defining dissimilarity measurements as follows:

- The squared Euclidean distance is measured by substituting the convex fucntion of the Bregman as $F\left(p\right)=\overset{d}{\underset{i=1}{\sum }}p_{i}^{2}=\langle p,q\rangle$, as shown in Figure 2.
- The Bregman divergence will lead to the KLD if the strictly convex function used is

$F\left(p\right)=\overset{d}{\underset{i=1}{\sum }}p_{i}logp_{i}$, which is defined as negative Shannon entropy. The KLD is defined as:

(9) $$KL(p||q)=\underset{s}{\sum }p\left(S=s\;\right)\log(\frac{p\left(S=s\right)}{q\left(S=s\right)})$$

In information-theoretic relative entropy, the Shannon entropy measures the uncertainty of a random variable by:

(10) $$H(p)\;=plog\frac{1}{p}$$

The KLD is equal to the cross-entropy of two discrete distributions minus the Shannon differential entropy [30]:

(11) $$KL(p||q)=\underset{s}{\sum }H^{x}(p(s)\;||q\left(s\right))-H\left(p\left(s\right)\right)$$

where $H^{x}$ is the cross-entropy:

(12) $$H^{x}(p(s)\;||q\left(s\right))=\underset{s}{\sum }p\left(s\right)log\frac{1}{q\left(s\right)}$$

Such a KLD has two major drawbacks. First, the output is undefined if *q* = 0 and *p* ≠ 0; and second, the KLD is not bound by terms of metric distance. To avoid these drawbacks and avoid the log(0) or to divide by 0, the authors in Reference [31] proposed a Jensen–Shannon divergence (JSD) dependent on the KLD as follows:

(13) $$JSD(p||q)=\frac{1}{2}\left(KL\left(p,\frac{p+q\;}{2}\right)+KL\left(q,\frac{p+q}{2}\right)\right)$$

**Figure 3:**
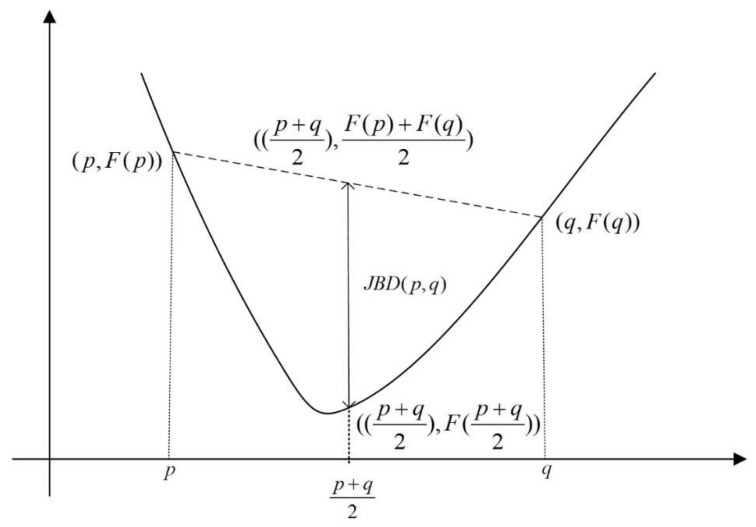
Interpreting the Jensen-Bregman divergence.

The JSD can be defined, is bound by an L1-metric, and is finite. In the same vein, the Bregman divergence (SD_F_) can be symmetrized as:

(14) $$SD_{F}(p||q)=\frac{1}{2}\left(D_{F}\left(p,\frac{p+q}{2}\right)+D_{F}\left(q,\frac{p+q}{2}\right)\right)\;=\frac{F(p)\;+F\left(q_{j}\right)}{2}-F\left(\frac{p+q_{j}}{2}\right)$$

where *p* represents the test point dataset, *q* represents the fingerprint dataset, and *j* represents the number of APs that the smartphone has received. Because F is a strictly convex function, the SD(p||q) equals zero if and only if *p* = *q*; the geometric interpretation for this is represented in Figure 3. For a positive definite matrix, the JBD is known as the Mahalanobis distance.

$$SD\left(p,q\right)=\frac{F\left(p\right)+F\left(q\right)}{2}-F\left(\frac{p+q}{2}\right)\;=\frac{2\langle Qp,p\rangle +2\langle Qq,q\rangle -2\langle Q\left(p,q\right),p+q\rangle }{4}\;=\frac{1}{4}\left(\langle Qp,p\rangle +\langle Qq,q\rangle -2\langle Qp,q\rangle \right)\;=\frac{1}{4}\langle Q\left(p-q\right)-p-q\rangle \;=\frac{1}{4}{\left|\left|p-q\;\right|\right|}_{Q}^{2}$$

Due to RSS variation and the hardware variance problem, the fingerprinting database of the offline phase was clustered by using a clustering algorithms technique. The k-means algorithm was proposed by Lloyd in 1957 [32], who is considered a pioneer in clustering methods. In general, the k-means was used to solve the vector quantization problem. k-means is an iterative clustering algorithm that works by choosing random data points (seeds) to be the initial centroid (cluster center); the points of each cluster are associated with the closest cluster center. Each cluster center is updated and reiterated until the difference between any successive calculation goes below the “loss function” or convergence is met. The squared Euclidean distance is used to minimize the intra-cluster distance that leads to the centroids. Lloyd [32] further proved that the iterative k-means algorithm monotonically converges to a local optima of the quadratic function loss (minimum variance loss). The cluster *C_i_*’s center *c_i_* is defined as follows:

(15) $$c_{i}=\arg\min\underset{p_{j}\epsilon c_{i}\;}{\sum }\|p_{j}-c_{i}\|$$

(16) $$=\arg\min\;AVG_{L_{2}^{2}\;}\left(C_{i},c\right)\;$$

(17) $$c_{i}=\frac{1}{\left|C_{i}\;\right|}\underset{p_{j}\epsilon c_{i}}{\sum }p_{j}$$

where *c_i_* denotes the center of the cluster *C_i_*, and $\left|C_{i}\right|$ denotes the cardinality of *C_i_*. In 2004, Reference [33] proposed a new clustering algorithm method, in which the k-means algorithm is modified by using the symmetric Bregman divergence. The minimum distance of the centroid of the point set has been defined as:

(18) $$c=\arg_{p}\min=\frac{1}{n}\;\sum_{i}SD_{F}\left(p,p_{i}\right)$$

(19) $$c_{R}^{F}=\arg_{c\in RP}\min\frac{1}{n}\;\overset{n}{\underset{i=1}{\sum }}SD_{F}(p_{i}||c)$$

(20) $$c_{L}^{F}=\arg_{c\in RP}\min\frac{1}{n}\;\overset{n}{\underset{i=1}{\sum }}SD_{F}(c||p_{i})$$

(21) $$c^{F}=\arg_{c\in RP}\min\frac{1}{n}\;\overset{n}{\underset{i=1}{\sum }}\frac{SD_{F}(c||p_{i})+\;SD_{F}(c||p_{i})}{2}$$

where $c_{R}^{F}$ and $c_{L}^{F}$ represent the right- and left-sided centroid, the centroid $c^{F}$ stands for the symmetrized Bregman divergence centroid, and *n* stands for the number of cells of the off-line database in each cluster.

## 5. Overall Structure of Proposed Positioning Algorithm

Designing an IPS by depending on fingerprinting-based locations is difficult because the environment suffers from inference and discrimination, which can lead to a heterogeneous RSS. As a result, depending on L_p_-norm or square Euclidean distance algorithms do not always lead to systems with high accuracy. For example, it was proved in Reference [7] that the concave-convex procedure can obtain higher accuracy than algorithms that depend on the square Euclidean distance such as the kNN and probabilistic neural network (PNN). In this section, we introduce the symmetric Hölder divergence. To measure the similarity between *p* and *q*, where *rhs* and *lhs* denote the right-hand side and left-hand side, respectively, one can use bi-parametric inequalities, i.e., one can use *lhs*(*p*,*q*) *≤* *rhs*(*p*,*q*), and a similarity can be measured by using the log-ratio gap:

(22) $$D\left(p:q\;\right)=-\log(\frac{lhs\left(p,q\right)}{rhs\left(p,q\right)})=\log(\frac{rhs\left(p,q\right)}{lhs\left(p,q\right)})\geq 0$$

The Hölder divergence between two values *p(x)* and *q(x)* is:

(23) $$D^{H}\left(p:q\;\right)=-\log(\frac{\int p{\left(x\right)}^{\gamma /\alpha }q{\left(x\right)}^{\gamma /\beta }dx}{{\left(\int p{\left(x\right)}^{\gamma }dx\right)}^{1/\alpha }{\left(\int q{\left(x\right)}^{\gamma }dx\right)}^{1/\beta }})$$

where γ represents the power of the absolute value Lebesgue integrable, α,β represents the conjugate exponents, and *p*(*x*) and *q*(*x*) are positive measures as scalar values. Hölder divergence suffers from the law of the identity of indiscernible (self-distance is not equal to zero if *p(x) = q(x)*), the triangle-inequality, and the symmetry. The Hölder divergence encapsulates both the one-parameter family of skew Bhattacharyya divergence and Cauchy–Schwarz divergence [34]. The Hölder divergence yields to the Cauchy–Schwarz divergence if we set γ, α,β = 2:

(24) $$D_{2,2\;}^{H}\left(p:q\right)=CS\left(p:q\right)∶=-\log(\frac{\int p\left(x\right)q\left(x\right)dx}{{\left(\int p{\left(x\right)}^{2}dx\right)}^{1/2}{\left(\int q{\left(x\right)}^{2}dx\right)}^{1/2}})$$

The Hölder divergence will yield to the skew Bhattacharyya divergence if we set γ=1:

(25) $$D_{\alpha ,1\;}^{H}\left(p:q\right)=B_{1/\alpha }\left(p:q\right)∶=-\log\left(\int p{\left(x\right)}^{1/\alpha }q{\left(x\right)}^{1/\beta }dx\right)$$

**Figure 4:**
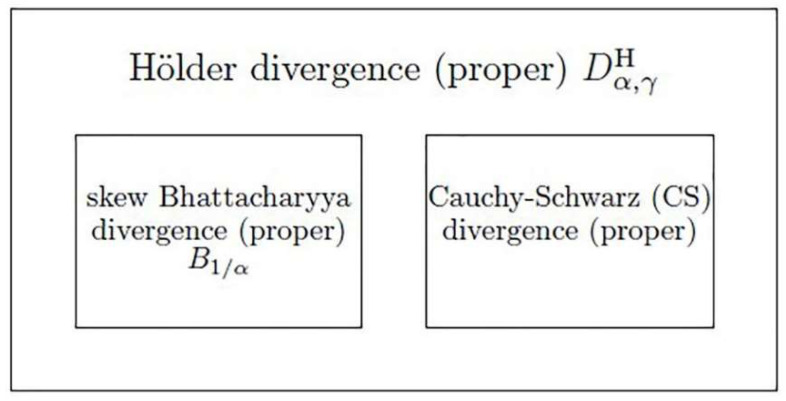
Hölder divergence encompasses the skew Bhattacharyya divergence and the Cauchy-Schwarz divergence.

The relationship between the divergence families is illustrated in Figure 4.

Similarly, for conjugate exponents *β* and *α*, the Hölder divergence satisfies:

(26) $$D_{\alpha ,\gamma }^{H}\left(p:q\right)=D_{\beta ,\gamma }^{H}\left(p:q\right)$$

The symmetrized Hölder divergence is:

(27) $$D_{\alpha ,1}^{H}\left(p:q\right)=\frac{1}{2}\left(D_{\alpha ,\gamma }^{H}\left(p:q\right)+D_{\alpha ,\gamma }^{H}\left(q:p\right)\right)$$

(28) $$=\frac{1}{2}\left[F\left(\gamma p\right)+F\left(\gamma q\right)-F\left(\frac{\gamma }{\alpha }p+\frac{\gamma }{\beta }q\right)-F\left(\frac{\gamma }{\beta }p+\frac{\gamma }{\alpha }q\right)\right]$$

**Figure 5:**
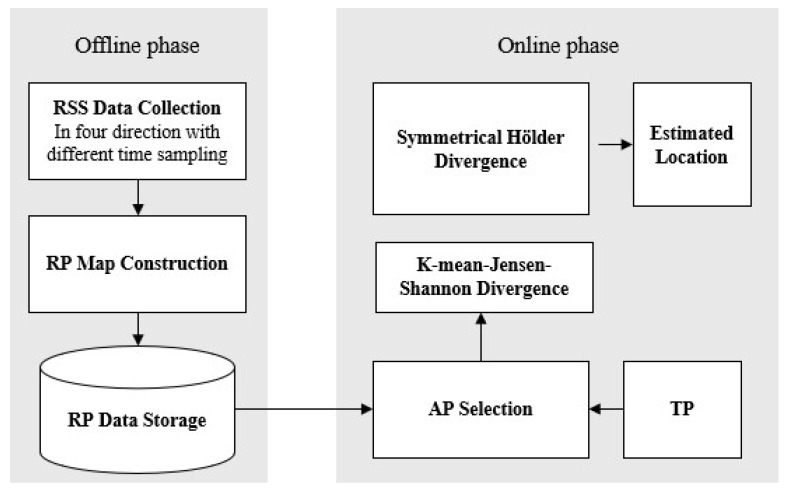
The offline and online stages of location WiFi-based fingerprinting architecture.

To improve the accuracy of the IPS, we proposed that sided and symmetrized Bregman centroids incorporate the symmetrized Hölder divergence. Furthermore, we introduce three different approaches to define the APs that will be used in the proposed algorithm, as shown in Figure 5.

∙ *Strongest APs (MaxMean)* [35]

Previous studies have proposed that the RSS be chosen based on the signal strength in the online phase, and that the same set of APs from the fingerprinting radio map be used in the calculations, with the assumption that the APs with the highest signal provide the highest coverage over time. However, the strongest AP scheme may not render a good criterion in our calculation.

∙ *Fisher Criterion:*

The Fisher criterion is a metric that is used to quantify the discrimination ability of APs across a fingerprinting radio map in four different orientations. The statistical properties of the RPs are used to determine the APs that will be used based on their performance. A score is pointed to each AP separately as [36]:

(29) $$\xi_{i}=\frac{\sum_{j=1\;}^{N}{\left(q_{j}^{i\left(o\right)}-\bar{q_{i}}\right)}^{2}}{\sum_{j=1}^{N}\Delta_{j}^{i\left(o\right)}}$$

(30) $$\bar{q_{i}\;}=\frac{1}{N}\overset{N}{\underset{j=1}{\sum }}q_{j}^{i}$$

The Fisher criterion proposes that APs with higher variance are less reliable to use in IPS calculations; the APs will be sorted with respect to their score, and those with high scores will be much more likely to be selected. However, Fisher criterion discrimination is only used in offline fingerprinting based-localization. If one or more APs are not available in the online phase, the Fisher criterion is not suitable to use.

∙ *Random Selection*

Unlike the above schemes, in which APs are selected based on some criteria, in random selection, the APs are selected arbitrarily without considering AP performance. This scheme has less computational complexity, as the matrix of the APs needs to be generated at different runs and does not need the variance to be calculated, as with the Fisher criterion.

## 6. Simulation and Implementation Results

**Figure 6:**
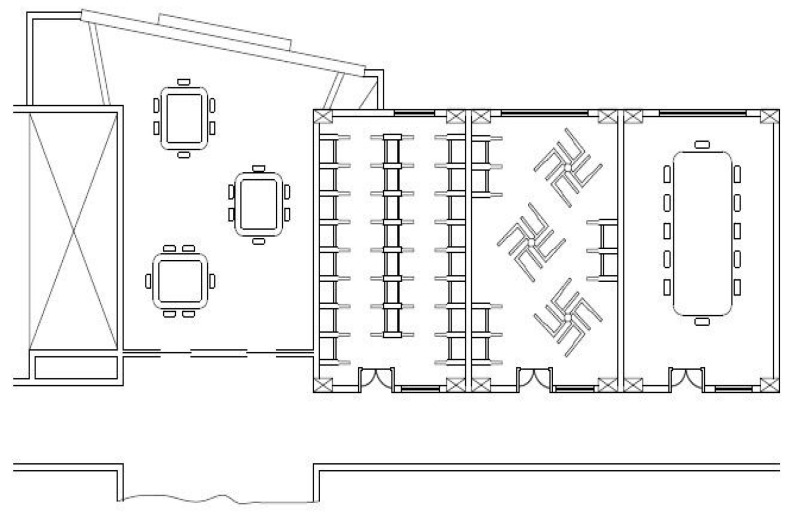
The layout used in the experimental work in the College of Engineering and Applied.

This section provides details on the proposed algorithms outlined in subsequent subsections. The RSS data were collected on the first floor of the CEAS at WMU with an area of interest map, as shown in Figure 6. A Samsung smartphone with operating system 4.4.2 (S5, Samsung Company, Suwon, Korea) was used to collect the RSS data. Furthermore, the proposed algorithms were implemented on an HP Laptop using Java software (HP, Beijing, China) with an Eclipse framework (Photon, IBM, NY, USA). Cisco Linksys E2500 Simultaneous Dual-Band Routers were used for the area of interest. The RSS value and MAC address of the WiFi APs were collected within a time frame of 1 s for 100 s over 84 RPs within an average grid of 1 m. At each RP, a total of 47 APs were detected throughout the area of interest.

**Figure 7:**
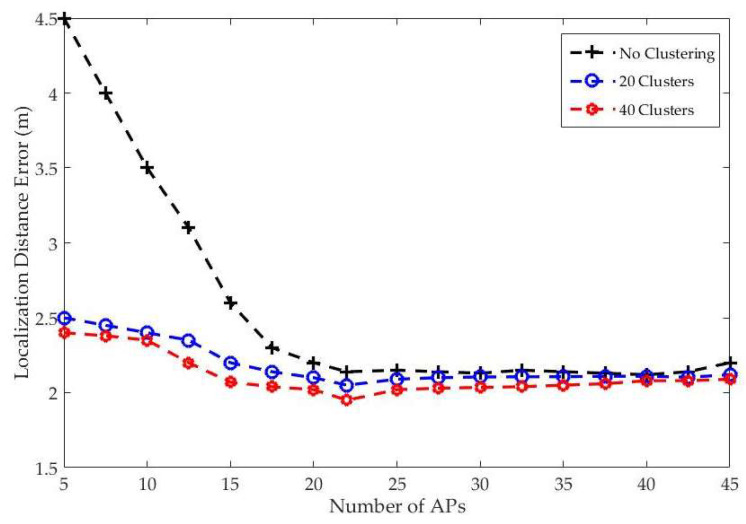
The implementation results of different number of clusters with respect to the average of the localization distance.

To evaluate the performance, online phase data were collected in varying environments on different days in 65 unknown locations with four repetitions as test points. The localization distance error was measured by calculating the Euclidean distance between the actual location of the testing point and the location that was estimated by the proposed algorithms. To reduce the RSS time variation, the k-means-Bregman divergence was used on the fingerprinting radio map to cluster the offline data. Figure 7 illustrates the effects of the clustering algorithms on localization distance error with the number of APs when five NNs are used. As shown in Figure 7, the localization distance error was decreased as the numbers of cluster increased, which reduced the area of interest that could improve object localization.

**Figure 8:**
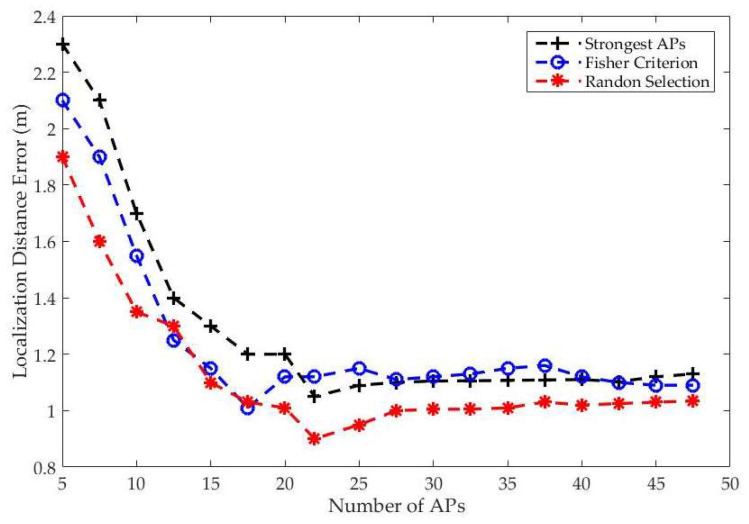
The implementation result of the average localization error under different AP selection schemes.

Figure 8 shows the localization distance error when a different AP selection scheme was used with the symmetrized Hölder divergence and k-mean-Bregman divergence, where the *y*-axis is the localization distance error and the *x*-axis is the number of APs. The Fisher criterion had the highest accuracy when the APs were less than 18, and the proposed random scheme achieved the next highest performance. The strongest AP scheme had a lower accuracy than the other schemes. In general, using more APs may not necessarily yield the lowest localization error. As shown in Figure 8, the best performance occurred when 22 APs were used; as the number of APs increased after that, the performance of the proposed systems decreased. Thus, we conclude that not only the number but also the selection scheme of APs can affect the IPS performance.

### Comparison to Prior Work

**Figure 9:**
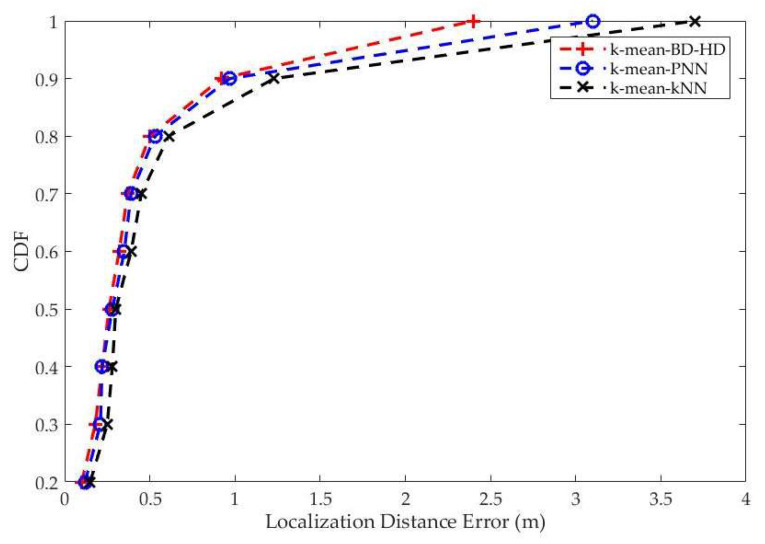
Experiment results: The Cumulative distribution function (CDF) of localization error when using 50 nearest neighbors.

The proposed fingerprint based-localization method is compared with prior fingerprinting approaches such as the kernel-based localization method, kNN. Figure 9 illustrates the corresponding cumulative probability distributions of the localization error for the three methods. In particular, the median error for the k-means-BD-HD was 0.92 m, 0.97 m for k-means-PNN, and 1.23 m for k-means-kNN.

As noticed, the proposed k-means-BD-HD method provides a 90th percentile error of 0.92 m, while for k-means-PNN it was 0.97 m, and for k-means-kNN it was 1.23 m.

## 7. Conclusions

IPSs incorporate the power of GPS and indoor mapping and have many potential applications that make them very important in modern life. For example, they can be used for healthcare services such as aiding people with impaired vision, and navigating unfamiliar buildings (e.g., malls, airports, subways). Several large companies, such as Apple, Google, and Microsoft, started a fund to initiate research on IPSs. Cluster methods can be used to reduce the impact of time variation by clustering the fingerprinting radio map into multiple partitions and then choosing the cluster that has the lowest distance error. A radio map fingerprint was developed in CEAS to investigate different localization algorithms and compare different approaches such as kNN and PNN. We proposed a symmetrical Hölder divergence, which uses statistical entropy that encapsulates both skew Bhattacharyya divergence and Cauchy–Schwarz divergence, and assessed their performance with different AP selection schemes. The results were quite adequate for the indoor environment with an average error of less than 1 m. the symmetrical Hölder divergence that incorporated the k-means-Bregman divergence had the highest accuracy when 25 clusters were used with 22 APs.

We are currently in the process of investigating the user position inside smaller clusters/areas and position prediction error distributions and quantifying the localization variation of WiFi signals distributed in space.
